# Emotions as Predictors of Life Satisfaction among University Students

**DOI:** 10.3390/ijerph17249462

**Published:** 2020-12-17

**Authors:** Óscar Gavín-Chocano, David Molero, Jose Luis Ubago-Jiménez, Inmaculada García-Martínez

**Affiliations:** 1 Department of Pedagogy, University of Jaén, 23071 Jaén, Spain; ogavin@ujaen.es (Ó.G.-C.); dmolero@ujaen.es (D.M.); 2 Department of Didactics of Musical, Plastic and Corporal Expression, University of Granada, 18012 Granada, Spain; 3 Department of Education, University of Almería, 04120 Almería, Spain; imartin@ual.es

**Keywords:** well-being, emotional intelligence, life satisfaction, academic performance, university students

## Abstract

Emotional management is a decisive factor in building stimulating environments for the comprehensive development of individuals. In this study, 338 students enrolled in education degrees (*n* = 338), with an average age of 22.88 years (±5.50), participated. The following instruments were used: Satisfaction with Life Scale (SWLS), Wong Law Emotional Intelligence Scale (WLEI-S), Trait Meta Mood Scale 24 (TMMS 24) and Emotional Quotient Inventory (EQi-C). The objective was to determine the complementarity of certain dimensions of EI that predict greater life satisfaction based on the multivariate statistics of structural equations. The multi-group model obtained good structural validity (χ^2^ = 103,729; RMSEA = 0.078; GFI = 0.917; CFI = 0.942; IFI = 0.943). In addition, significant correlations were found between life satisfaction and all dimensions were included in the emotional intelligence instruments used (*p* < 0.01). In terms of gender, we found that women had higher scores in all EI dimensions, in contrast to life satisfaction, where men had higher scores. The findings suggest the importance of working emotions in future educators to become satisfied and effective professionals.

## 1. Introduction

The university stage determines how learning and practical experiences, sequenced over time, are consolidated in a specific physical space that is capable of reproducing a professional model equipped with different resources and skills to meet different social demands [1]. These demands form part of the educational components of the 21st century, effectively linked to the acquisition of cognitive and emotional skills to ensure that university students become competent to face many challenges, which may be complex and stressful sometimes [2,3].

Different studies have analyzed the factors that contribute to the protection and well-being of individuals in the university context through the complementarity of cognitive and social-emotional skills. This was done to not only measure issues related to the learning outcomes generated by the university teaching activity, but also to improve features related to the individuals’ health [4,5], with academic performance [6], with the benefits of Emotional Intelligence (hereinafter EI), or as a resource to establish positive relationships, cooperative work, conflict resolution skills and positive thinking [7,8] and its relationship with context [9]. Evidence suggests that the proper management of emotional competencies, as well as their learning and development, may act as a preventive factor against negative emotional charge [10], assuming a key role in managing challenge situations properly [11], capable of promoting behaviors and prioritizing options that encourage decision making [12].

The concept of EI was developed theoretically in 1990, as one’s ability to perceive, understand and regulate one’s own emotions and those of others adaptively [13,14]. Then, it appears to be a multidimensional construct related to cognitive and emotional activity alongside, diversifying different conceptual models to guide this link in carrying out daily activities in different coexistence contexts [15] and it enhances the understanding about what emotional and intellectual qualities people hold [4].

Once it had been overcome, the debate regarding different criteria when establishing a unique and consistent notion of EI, different evaluation models and instruments emerged to advance over the essence of the construct itself [16]. Nowadays, the most commonly agreed upon conceptual categorization in EI distinguishes between capability model, measured through maximum performance tests and based on information processing, and mixed model, measured through self-report questionnaires [17]. The first one, called the capacity model [18], is focused on the ability to process information based on emotions to solve conflicts adaptively [16,19], and its description is relevant for the understanding of internal processes and the acquisition of emotional competencies [10,13,20,21]. A second approach, the mixed model [22,23], combine mental abilities with personality features, and it is defined as the set of emotional capacities, personal and interpersonal motivations that will condition the way of interacting when faced with external demands and pressures [24].

This study focuses its content on the mixed model, as it is one of the measures that has demonstrated the greatest theoretical and empirical soundness over the years, especially when it relates to other factors such as life satisfaction [25]. The value of personal life satisfaction include aspects related to personal and emotional development, lifestyle, coping strategies and achieving personal goals, placing positive feelings above negative ones [26,27]. Emotional feelings are essential to the individual’s expectations in order to manage success or failure in different life conditions [28,29]. Different studies find this distinction useful to understanding life satisfaction, taking into account the effective and emotional dimension in the academic context and its relationship with EI [30,31,32,33]. That is, university students with high levels of EI will be characterized by the effective use of cognitive and emotional competencies as adaptive skills. However, those who do not effectively perceive their own and others’ emotions will not be able to face stressful situations and will be more dissatisfied [4]. Thus understood, the acquisition of emotional competencies will be decisive not only academically, but also in different life situations [34].

Taking into account these considerations, this investigation focuses its attention on the complementarity of several evaluation instruments of EI (WLEIS-S, TMMS-24 and EQi-C), as predictive values to increase life satisfaction. This research provides continuity to previous studies that show significant positive relationships between some of the variables used with university students [35,36].

Therefore, this study is justified by the importance for future teachers to develop and work on emotional intelligence and psychosocial factors within their training period, as well as providing support for their subsequent professional development, and characterized by high levels of stress. Thus, the hypothesis is based on the fact that high levels of EI for future teachers will improve their quality of life. At the same time, the objectives proposed in this investigation, in general, are: (a) to analyze the existence of significant correlations between the dimensions of the EI instruments (WLEIS-S, TMMS-24 and EQi-C) and life satisfaction (SWLS), respectively; (b) to analyze the relationship between the dimensions of the instruments considered (SWLS, WLEIS-S, TMMS-24 and EQi-C) and the sociodemographic variables of age and university context; and (d) to study the effect of the variables of EI (WLEIS-S, TMMS-24 and EQi-C) and life satisfaction (SWLS) with the sociodemographic variable gender, using a multi-group structural equation model (SEM).

## 2. Materials and Methods

This descriptive study is based on a non-experimental, correlation-based, quantitative cross-sectional analysis. According to these criteria, longitudinal, comparative and score reliability measures are established, through Cronbach’s alpha and Omega coefficient calculation [37], also called Jöreskog Rho [38].

### 2.1. Participants

The sample is made up of 338 students of education degrees, belonging to the Universities in Jaén and Granada (Spain). The selection of the participants was based on a convenience sampling, not a probabilistic causal one, where the resulting sample was those individuals who accessed it voluntarily. The distribution by sex was the following: 280 women (82.84%) and 58 men (17.16%), coinciding with the predominant proportion in the education degrees of the Spanish universities. The age range is between 18 and 55 years, with an average age of 22.88 years (±5.70). The distribution by university context was 234 students from the University of Jaén (69.23%) and 104 students from the University of Granada (30.77%).

### 2.2. Instrument

The present study was developed around four instruments validated in the Spanish context, all of them renowned. The intention was to use valid and reliable instruments to measure the multidimensional character of the variables intended to be analyzed, i.e., life satisfaction and EI.

– Satisfaction with Life Scale. The Satisfaction with Life Scale -SWLS- was used to evaluate life satisfaction [39]. In particular, the five-item version of the Life Satisfaction Scale of Vázquez, Duque and Hervás [40] was used. The scale in the Spanish version reports an internal consistency of α = 0.82. The reliability of the scale scores obtained in our study is α = 0.80 and the Omega coefficient **ω** = 0.79.
– WLEIS-S. The Wong Law Emotional Intelligence Scale (WLEI-S) instrument, in its Spanish version [41], was used to measure EI. It is based on the Wong and Law WLEIS EI scale [42], and it includes 16 items and 4 dimensions: Intrapersonal Perception (Evaluation of own emotions), Interpersonal Perception (Evaluation of the emotions of others), Assimilation (Use of emotions) and Emotional Regulation. A Likert-type scale of 7 points (1 to 7 points) was used, the reliability of the scores for the variable “Evaluation of own emotions” ranging from α = 0.77 and **ω** = 0.76; α = 0.80 and **ω** = 0.76; for “Evaluation of the emotions of others”; “Use of emotions” with indexes of α = 0.78 and **ω** = 0.80; and α = 0.78 and **ω** = 0.75 for “Emotional regulation”.
– Trait Meta-Mood Scale-24-. The Trait Meta-Mood Scale-24 (TMMS-24), by Fernández-Berrocal, Extremera and Ramos [43], was used to measure EI. Its original version is the one developed by Salovey, Mayer, Goldman, Turvey and Palfai [12]. This tool has been used in many social science research contexts. The reliability obtained in our study is of α = 0.80 and **ω** = 0.74 for the dimension “Attention”; α = 0.77 and **ω** = 0.84 for “Clarity”; and for “Repair” some indexes of α = 0.78 and **ω** = 0.86.
– Emotional Quotient Inventory. The Spanish adaptation of the EQi-C scale of Bar-On [22], by López-Zafra, Pulido-Martos and Berrios [44], was used to measure the EI. The reliability for each subscale of the EQi-C is shown in our sample as α = 0.76 and **ω** = 0.65 for “Interpersonal”; α = 0.72 and **ω** = 0.64 for “Adaptability”; α = 0.84 and **ω** = 0.72 for “Stress Management”; and α = 0.77 and **ω** = 0.70 for “Intrapersonal”.

### 2.3. Procedure

Contact with the participants was made possible by professors who taught courses at the Faculties of Education from the Universities of Granada and Jaén. After explaining the purpose of the research, they were asked to inform and ask their students if they wanted to participate in the research. The potential participants were notified of the process to be followed, confidentiality and the anonymity of the evidence collected. For the administration of the instruments, we provided the link to them, using the Google Form TM tool, in order to assist them in filling out the instruments using their mobile devices. The filling-in was carried out during school hours, providing them the possibility of solving any possible doubts they may have during the filling-in process. Likewise, the codes and ethical guidelines of the Declaration of Helsinki [45] were followed.

### 2.4. Data Analysis

To achieve a better adjustment in each of the tests, the data were transformed according to their factorial load [46]. Descriptive statistics (means and standard deviations) were obtained, analyzing the reliability and internal consistency of each instrument through Cronbach Alpha and Omega coefficients. We worked with the weighted sum of each variable, overcoming the limitations that could affect the proportion of the variance [47] and the correlation between the resulting scores in each of the dimensions. Next, we performed a mean difference analysis according to age and university context with the Mann-Whitney U test of mean difference for non-related samples. Non-parametric tests were used as the assumption of normality was not fulfilled in all cases based on the data obtained in the Kolmogorov-Smirnov test (*n* > 50 cases). In addition, the effect size was reported in the analyses performed. Finally, a multi-group structural equation model (SEM) was developed for the sociodemographic variable gender, in order to evidence the existence of significant differences between all the variables contained in the instruments. In all cases, a 95% confidence level (significance *p* < 0.05) was used, employing the IBM SPSS Statistics 24.0TM (IBM, Chicago, IL) and AMOS 25, to obtain the results of the tests indicated above.

## 3. Results

This section may be divided by subheadings. It should provide a concise and precise description of the experimental results, their interpretation as well as the experimental conclusions that can be drawn.

### 3.1. Relationship between Life Satisfaction and Emotional Intelligence

Table 1 shows the scores of the correlation matrix (Spearman Rho, since it is a non-normal distribution), descriptive statistics (mean and standard deviation), reliability analysis (Cronbach alpha and Omega coefficient), and provides adequate reliability rating in general.

Analyzing each of the dimensions, statistically significant relationships between life satisfaction and all the dimensions included in the EI instruments (WLEIS-S, TMMS-24 and EQi-C) were observed, with the highest correlation established with the use of emotion dimension (r(334) = 0.56; *p* < 0.01). Similarly, there were significant relationships in most of the variables of EI, where the highest correlation is established between the appraisal of one’s own emotions and clarity (r(334) = 0.84; *p* < 0.01); use of emotion and repair (r(334) = 0.76; *p* < 0.01); and appraisal of others’ emotions and interpersonal (r(334) = 0.61; *p* < 0.01). We highlight the significant correlations in the opposite direction between the EI variable of the EQi-C stress management instrument and the rest of the variables, being the most important one established with the regulation of emotion (r(177) = −0.44; *p* < 0.01).

### 3.2. Differences According to Socio-Demographic Variables

We used the non-parametric Mann-Whitney U test for two independent samples to analyze the differences in terms of the sociodemographic variable age (<25 years vs. >25 years) (See Table 2). The results indicate the statistically significant relationship between life satisfaction and age (Z = −2.758; *p* > 0.01), in favor of the younger ones. There are no significant differences in the other dimensions of the EI WLEIS-S, TMMS-24 and EQi-C instruments in relation to age (Z < 2.0; *p* > 0.05 ns). To calculate the effect size, we obtained the value of r [*r* = Z/√*n*]. The effect size was small in all cases (r < 0.2), according to the Cohen criteria.

Regarding the university context (See Table 3), the results point out that there are significant differences regarding the life satisfaction variable (Z = −4.3342; *p* > 0.01), with higher scores for students from the University of Jaén. There are also significant differences in three of the dimensions of EI included in the WLEIS-S instrument in relation to the university context. Here, it highlight the appraisal of one’s own emotions variable (Z = −3.547; *p* > 0.01). Similarly, we found significant differences in all the variables of the EI (TMMS-24) in relation to the university context, highlighting the clarity dimension (Z = −3.158; *p* > 0.01). Finally, we found a significant relationship between the adaptability variable of the EI instrument (EQi-C) and the university context (Z = −3.291; *p* > 0.01). The effect size was small in all cases.

### 3.3. Multilevel Regression Study

With the aim of exploring and quantifying the predictive capacity of the variables of the EI on life satisfaction, a multiple regression analysis was carried out, discarding, retrospectively, those variables that did not enter the regression model, verifying the absence of multicollinearity problems (tolerance values being <0.20; IVF > 4.00), our values being between 1.321 and 3.934. The results of the Durbin-Watson test indicate that there is an independence of mistakes, with a value of 1.589. As it was between 1 and 3, we accepted the assumption.

The dimension included in the regression model explains 41.0% of the variance, with the EI variable evaluation of one’s own emotions as the best predictor of life satisfaction (R = 0.640; R2 Corrected = 0.403; F = 33.248), with the t value significant in the remaining variables (Table 4).

### 3.4. Multi-Group or Multi-Sample Structural Equation Model

The model fit was tested using Chi-square (χ^2^), goodness-of-fit index (GFI) and approximation mean square error (RMSEA) as absolute fit measures. Goodness-of-fit index (AGFI), Tucker-Lewis Index (TLI) and comparative goodness-of-fit index (CFI) as measures of incremental fit. The Chi-square ratio (χ^2^) on levels of freedom (CMIN/GL) and the Akaike Information Criterion (AIC), as measures of parsimony adjustment [48].

First, the validity and fit of the established model was checked from the data obtained in the hierarchical regression analysis, displaying a significant associated Chi-square value (χ^2^) (χ^2^ = 103.729; gl = 34; *p* = 0.001). However, this statistic is sensitive to sample size and should be interpreted with caution. Therefore, different studies recommend using other indicators to assess the model fit [49]. Among the most widely used are the goodness-of-fit index (GFI), which has a value of 0.917, representing an acceptable model fit, and the comparative fit index (CFI), which has a value of 0.942. The corrected goodness-of-fit index (AGFI) value was higher than 0.85, which also suggests a good fit.

Finally, the root mean square error (RMSEA) shows an anticipated adjustment with the total value of the population, being less than 0.08 for the established parameters. The values of this index were proposed by Steiger and Lind [50], who suggested compensating the effect of the complexity of the model dividing by the number of levels of freedom to test the model. Values lower than 0.08 are indicative of a good fit, being in our case 0.078. Consequently, the model fit is acceptable in relation to the data obtained. Given the lack of balance of our sample regarding sex, consistent with the proportions found for the Spanish university population from education degrees, we decided to present two models.

**Figure 1:**
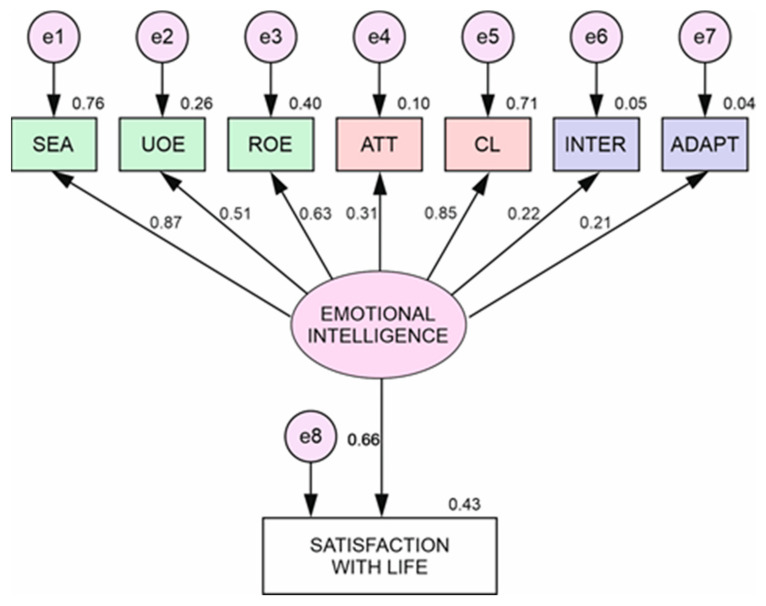
Multi-group structural equation model for men.

Figure 1 shows the standardized weights between each of the variables for men, setting a significance level of 0.005 (5% probability of error), for the indicators with a greater weight of regression of the variables below this value (See Table 5), corresponding to the EI: evaluation of own emotions (5.466), clarity (5.289), use of emotions (3.516) and attention (2.103). Additionally, this was between life satisfaction and EI (4.227) and optimism (3.436).

**Figure 2:**
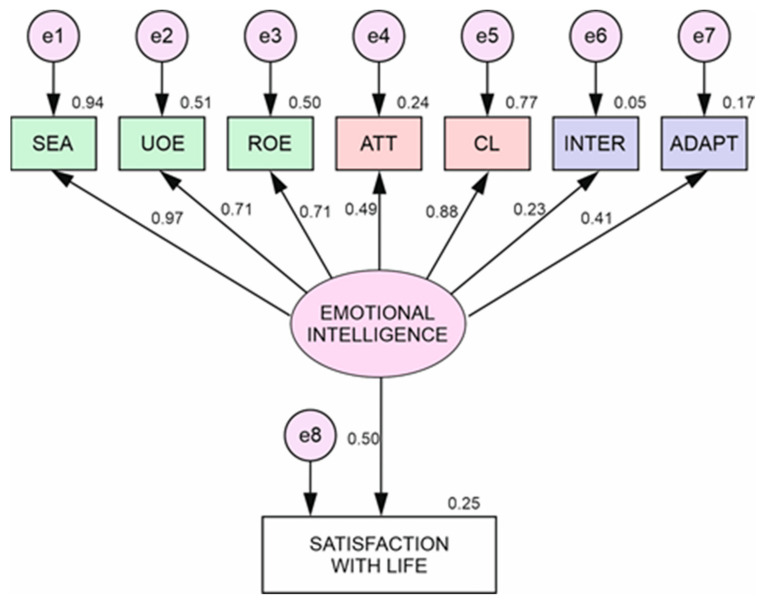
Multi-group structural equation model for women.

Figure 2 illustrates the standardized weights of saturation corresponding to women (see Table 6), establishing the indicators with the greatest weight of regression for the variables of EI: evaluation of own emotions (9.054), clarity (8.858), use of emotions (8.081), emotional regulation (8.062), attention (6.471), adaptability (85.778) and interpersonal (3.498). Likewise, between life satisfaction and EI (3.385).

We found higher values for women than men in almost all the variables of EI, highlighting “evaluation of own emotions” (S.R.W.= 0.97) and “use and regulation of emotions” (S.R.W.= 0.71). In contrast, the data obtained between EI and life satisfaction are higher for men than for women (S.R.W. = 0.66). These results should be carefully considered due to the high number of women in our sample.

## 4. Discussion

The main objective of this study was to determine the complementarity of the EI instruments (WLEIS-S, TMMS-24 and EQi-C) as predictors of higher life satisfaction in students of education degrees, belonging to the Universities of Jaén and Granada (Spain). Results generally agree with other studies [4,6,10]. They highlight the positive impact of some dimensions of EI on life satisfaction, with differences according to gender, despite the distributional asymmetry (more women than men), which may affect the results obtained. In this regard, the distribution of the global trend in education-related studies, with a greater number of women, should be taken into account.

First, the reliability of each of the instruments was verified using the Cronbach alpha and subsequently the Omega coefficient, the last is the most appropriate estimate when there is a disparity in the factorial load of each item (Tau-Equivalence), when working with the weighted sum of each variable and overcoming the limitations that could affect the proportion of the variance [47,48,51].

According to the first objective, the results revealed significant correlation between life satisfaction and each of the EI dimensions. The most valuable correlation was the one established with the use of emotions (UOE). Our study revealed the complementarity of each of the dimensions of EI to achieve greater psychological well-being and life satisfaction [16,17], that it is consistent with other studies. In other words, perception and emotional management are determining factors in regulating a positive state of mind [52]. As our results suggest, the learning of emotional skills can be decisive in adaptive problem solving [10]. While the background found a direct link between each of the dimensions of EI and life satisfaction among university students, the findings presented in this study confirm the relevance of training proposals based on emotions in educational processes [4,7].

The emotional competencies will enable university students to not only commit themselves to their academic activities, but also to create a strong base on which to sustain their performance in different aspects of their lives, in order to face adverse situations successfully [53].

In relation to the second objective, to establish the existence of significant differences between the dimensions of the instruments considered and the sociodemographic variables, we have found significant differences between life satisfaction with age, in favor of the younger ones. With regard to the relationship between each of the variables of the EI with age, there are no significant differences, and they are slightly higher for younger people. It is possible that younger students are more idealistic, defend their future projection and desire to improve, showing more confidence in themselves, which could translate into greater life satisfaction [54]. Based on previous studies that confirm these results [55], it can be seen that life satisfaction increases when students show more emotional competencies [16].

Regarding the relationship between life satisfaction, EI and the socio-demographic variable university context, significant relationship between some of the variables measured and the context in which they carry out their studies was noted, with slightly higher scores in favor of the University of Granada. The quality of the teaching-learning environment is likely to significantly affect the development of features related to the development of emotional competencies [10,56]. However, most students perceive greater life satisfaction during their university period, noting a high grade of well-being in the variables analyzed. However, the background of the people in our study, access to resources and environments that could affect the results obtained should be taken into consideration [2]. This study opens up the possibility of establishing a strategic plan to consolidate training in emotional competencies, with the aim of increasing educational quality in order to improve student satisfaction [4].

Finally, to determine which EI variables predict greater life satisfaction, a multi-level regression analysis was performed, rejecting those dimensions with non-significant values. In our case, the EI variables were: evaluation of one’s emotions, use of emotions, emotional regulation, attention, clarity, interpersonal and adaptability. According to Kahn [57], the multi-variate hierarchical regression model is one of the most common techniques that enable verification in advance of variables that are significant in relation to the established model.

This approach is reasonable, based on these criteria. Then, a multi-group analysis was developed with the multivariate statistical technique of structural equations, to corroborate what was reported in the regression analysis, presenting the model with a good fit on the effect of EI on life satisfaction according to sex, whereby women are better qualified than men in the variables: evaluation of their own emotions, use of emotions, emotional regulation and clarity. These results are consistent with other studies that point to higher EI scores for women from different environmental, cultural and social contexts [16,58]. Analyzing previous research that corroborates these data [28], we can point out that women are better capable of attending and recognizing their emotions in order to face day-to-day situations effectively [6,15].

Furthermore, the EI variables (interpersonal and adaptability) showed higher values for men than for women. This data could justify the stereotyped view that remains in some contexts, where women hold back and do not openly show their emotions [9]. It may be that evidence is conditioned by the higher number of women in the sample, which would make it difficult to extend the results to other contexts. Points that also highlight the reasons for horizontal segregation when students choose university studies, namely the Humanities; Experimental Sciences; Social and Legal Sciences; and Health Sciences, which are mostly chosen by women [59]. Finally, there are non-significant values in the dimensions of EI care, interpersonal and adaptability, which are higher for women. According to other studies [26], we note that women are more sensitive to better recognizing emotions and to face day-to-day situations adaptively [16,19].

## 5. Conclusions

During this investigation, the results obtained have been detailed, based on the complementarity of some dimensions of the EI instruments (WLEIS-S, TMMS-24 and EQi-C) as predictors of greater life satisfaction. The contributions presented are useful for several reasons. Firstly, to find out what psychological and emotional qualities people acquire within the university context [7]. Secondly, to analyze the positive features regarding the use, emotional regulation and adaptability as the basis for action aimed at promoting support processes, strengthening different skills to prevent risk behaviors [25]. From this perspective, we understand that EI may favor greater life satisfaction, offering creative solutions to adverse situations. Therefore, health, social, academic and professional success, as well as overall quality of life are positively considered [60]. Finally, these findings show that EI is a relevant indicator of vital satisfaction, so it is essential to promote actions within the university context [9].

Despite the evidence reported, it is necessary to point out some limitations. The subjective functionality of the self-reporting instruments may condition the data in terms of adjustments of social desirability. In addition, the sample size, as well as gender differences, its heterogeneity and geographical limitation do not permit extending the results to other contexts, so it would be advisable to explore the factorial invariance of the assessment instruments in further research in order to verify whether they are cross-cultural measures [28]. This limitation makes the findings found to be treated with caution pending their contrast in other studies and contexts.

## Figures and Tables

**Table 1:** Internal consistency, mean, standard deviation and Spearman’s Rho correlation of the variables life satisfaction and emotional intelligence.

Dimension	*α*	ω	*M* (*SD*)	SV	SEA	OEA	UOE	ROE	ATT	CL	REP	INTER	ADAP	STR	INTRA
SWL	0.8	0.79	19.39 (±4.9)	-	0.50 **	0.11 *	0.56 **	0.48 **	0.12 *	0.45 **	0.44 **	0.14 **	0.17 **	−0.18 **	0.25 **
SEA	0.77	0.76	11.30 (±2.3)		-	0.40 **	0.67 **	0.66 **	0.43 **	0.84 **	0.57 **	0.33 **	0.37 **	−0.14 **	0.28 **
OEA	0.8	0.76	18.17 (±2.7)			-	0.22 **	0.33 **	0.39 **	0.40 **	0.24 **	0.61 **	0.38 **	−0.17 **	0.19 **
UOE	0.78	0.8	14.39 (±3.4)				-	0.62 **	0.30 **	0.56 **	0.76 **	0.22 **	0.32 **	−0.26 **	0.25 **
ROE	0.78	0.75	13.36 (±3.1)					-	0.41 **	0.61 **	0.62 **	0.25 **	0.40 **	−0.44 **	0.17 **
ATT	0.8	0.74	26.40 (±5.3)						-	0.53 **	0.36 **	0.41 **	0.25 **	−0.05	0.08
CL	0.77	0.84	30.31 (±6.0)							-	0.52 **	0.34 **	0.35 **	−0.13 *	0.24 **
REP	0.78	0.86	26.16 (±5.7)								-	0.20 **	0.39 **	−0.25 **	0.09
INTER	0.76	0.65	29.33 (±3.2)									-	0.36 **	−0.03	0.26 **
ADAP	0.72	0.64	18.23 (±2.9)										-	−0.11 *	0.22 **
STR	0.84	0.72	21.50 (±7.0)											-	−0.14 *
INTRA	0.77	0.7	26.78 (±3.4)												

Note: (1) Mean = *M*; Standard deviation = *SD*; Life satisfaction = SWL; Self Emotion Assessment = SEA; Other’s Emotions Assessment = OEA; Use of Emotions = UOE; Emotional regulation = ROE; Emotional Intelligence Attention = ATT; Clarity = CL; Repair = RE; Interpersonal Emotional Intelligence = INTER; Adaptability = ADAP; Stress management = STR; Intrapersonal Emotional Intelligence = INTRA. (2) * = *p* < 0.05; ** = *p* < 0.01.

**Table 2:** Mean differences according to age (U of Mann-Whitney).

Variables		<25 Years *M* (*SD*)	>25 Years *M* (*SD*)	*Z*	*p*	Effect Size (*r*)
SWLS	SWL	19.81 (±4.7)	17.86(±4.9)	−2.758	0.006 **	0.15
WLEIS-S	SEA	11.20 (±2.2)	11.73 (±2.4)	−1.856	0.064	0.1
	OEA	18.19 (±2.7)	18.02 (±2.8)	−0.373	0.709	0.02
	UOE	14.25 (±3.5)	14.91 (±3.2)	−1.214	0.225	0.06
	ROE	13.49 (±3.1)	12.73 (±3.3)	−1.257	0.209	0.06
TMMS-24	ATT	26.48 (±5.2)	25.87 (±6.0)	−0.686	0.493	0.03
	CL	30.13 (±5.9)	30.99 (±6.7)	−1.42	0.156	0.07
	REP	25.81 (±5.8)	27.37 (±4.9)	−1.666	0.096	0.09
EQi-C	INTER	29.47 (±3.1)	28.70 (±3.8)	−1.211	0.226	0.06
	ADAP	18.16 (±3.0)	18.46 (±2.7)	−0.427	0.67	0.01
	STR	21.32 (±6.9)	21.95 (±7.0)	−0.263	0.792	0.04
	INTR	26.77 (±3.2)	26.71 (±4.3)	−1.267	0.205	0.06

Note: (1) Mean = *M*; Standard deviation = *SD*; Life satisfaction = SWL; Self Emotion Assessment = SEA; Other’s Emotions Assessment = OEA; Use of Emotions = UOE; Emotional regulation = ROE; Emotional Intelligence Attention = ATT; Clarity = CL; Repair = RE; Interpersonal Emotional Intelligence = INTER; Adaptability = ADAP; Stress management = STR; Intrapersonal Emotional Intelligence = INTRA. (2) The effect size is expressed with Cohen value. (3) ** = *p* < 0.01.

**Table 3:** Mean differences according to the University (U of Mann-Whitney).

Variables		University of Jaén *M* (*SD*)	University of Granada *M* (*SD*)	*Z*	*p*	Effect Size (*r*)
SWLS	SWL	20.20 (±4.5)	17.78(±5.0)	−4.334	0.001 **	0.23
WLEIS-S	SEA	11.53 (±2.3)	10.78 (±2.2)	−3.547	0.006 **	0.19
	OEA	18.40 (±2.6)	17.62 (±2.7)	−2.437	0.015 *	0.13
	UOE	14.47 (±3.5)	14.17 (±3.2)	−0.863	0.388	0.04
	ROE	13.65 (±3.1)	12.67 (±3.2)	−2.467	0.014 *	0.13
TMMS-24	ATT	26.76 (±5.3)	25.51 (±5.5)	−2.123	0.034 *	0.11
	CL	30.93 (±5.7)	28.87 (±6.6)	−3.158	0.002 **	0.17
	REP	26.47 (±5.7)	25.27 (±5.6)	−2.034	0.042 *	0.11
EQi-C	INTER	29.50 (±3.2)	28.94 (±3.3)	−1.502	0.133	0.08
	ADAP	18.56 (±2.8)	17.44 (±3.1)	−3.291	0.001 **	0.18
	STR	21.12(±6.8)	22.16 (±7.4)	−1.211	0.226	0.06
	INTR	26.79 (±3.4)	26.70 (±3.5)	−0.417	0.676	0.02

Note: (1) Mean = *M*; Standard deviation = *SD*; Life satisfaction = SWL; Self Emotion Assessment = SEA; Other’s Emotions Assessment = OEA; Use of Emotions = UOE; Emotional regulation = ROE; Emotional Intelligence Attention = ATT; Clarity = CL; Repair = RE; Interpersonal Emotional Intelligence = INTER; Adaptability = ADAP; Stress management = STR; Intrapersonal Emotional Intelligence = INTRA. (2) The effect size is expressed with Cohen value. (3) * = *p* < 0.05; ** = *p* < 0.01.

**Table 4:** Multilevel regression analysis, criteria variable: life satisfaction.

Criteria Variable	*R*	*R* ^2^	*R*^2^ Corrected	*F*	Predicting Variables	*β*	*t*
Satisfaction with life	0.64	0.41	0.403	33.248			
					SEA	0.508	10.769 **
					UOE	0.4	7.091 **
					ROE	0.199	3.351 **
					ATT	0.184	3.667 **
					CL	0.215	3.563 **
					INTER	0.151	2.798 **
					ADAP	0.183	3.406 **

*Note:* (1) Self Emotion Assessment = SEA; Other’s Emotions Assessment = OEA; Use of Emotions = UOE; Emotional regulation = ROE; Emotional Intelligence Attention = ATT; Clarity = CL; Interpersonal Emotional Intelligence = INTER; Adaptability = ADAP. (2) ** = *p* < 0.01.

**Table 5:** Regression weights and standardized regression weights for men.

Relationships between Variables			Estimations	R.W. E.E.	C.R.	*p*	S.R.W. Estimations
SWL	<---	EI	0.673	0.159	4.227	***	0.658
SEA	<---	EI	0.496	0.091	5.466	***	0.871
UOE	<---	EI	0.393	0.112	3.516	***	0.513
ROE	<---	EI	1				0.63
ATT	<---	EI	0.274	0.13	2.103	***	0.309
CL	<---	EI	0.711	0.134	5.289	***	0.845
INTER	<---	EI	0.165	0.107	1.54	0.124	0.216
ADAPT	<---	EI	0.123	0.084	1.471	0.141	0.206

Note: (1) Regression weights= R.W.; Standardized regression weights= S.R.W.; Error estimation= E.E.; Critical ratio= C.R. (2) Emotional intelligence = EI, Life satisfaction = SWL, Evaluation of own emotions = SEA, Use of emotions = UOE, Emotional regulation = ROE, Attention = ATT, Clarity = CL, Interpersonal = INTER, Adaptability = ADAP. (3) *** *p* < 0.01.

**Table 6:** Regression weights and standardized regression weights for women.

Relationships between Variables			Estimations	R.W. E.E.	C.R.	*p*	S.R.W. Estimations
SWL	<---	EI	0.496	0.147	3.385	***	0.497
SEA	<---	EI	1.174	0.13	9.054	***	0.968
UOE	<---	EI	1.312	0.162	8.081	***	0.711
ROE	<---	EI	1.129	0.14	8.062	***	0.707
ATT	<---	EI	0.665	0.103	6.471	***	0.488
CL	<---	EI	1.398	0.157	8.858	***	0.877
INTER	<---	EI	0.301	0.086	3.498	***	0.227
ADAPT	<---	EI	0.517	0.09	5.778	***	0.414

Note: (1) Regression weights= R.W.; Standardized regression weights= S.R.W.; Error estimation= E.E.; Critical ratio= C.R. (2) Emotional intelligence = EI, Life satisfaction = SWL, Evaluation of own emotions = SEA, Use of emotions = UOE, Emotional regulation = ROE, Attention = ATT, Clarity = CL, Interpersonal = INTER, Adaptability = ADAP. (3) *** *p* < 0.01.
